# A Global Analysis of the Polygalacturonase Gene Family in Soybean (*Glycine max*)

**DOI:** 10.1371/journal.pone.0163012

**Published:** 2016-09-22

**Authors:** Feifei Wang, Xia Sun, Xinyi Shi, Hong Zhai, Changen Tian, Fanjiang Kong, Baohui Liu, Xiaohui Yuan

**Affiliations:** 1 Northeast Institute of Geography and Agroecology, Key Laboratory of Soybean Molecular Design Breeding, the Chinese Academy of Sciences, Harbin, 150081, China; 2 University of Chinese Academy of Sciences, Beijing, 100049, China; 3 School of Computer Science and Technology, Heilongjiang University, Harbin, 150080, China; 4 School of Life Sciences, Guangzhou University, Guangzhou, 510006, China; Chinese University of Hong Kong, HONG KONG

## Abstract

Polygalacturonase is one of the pectin hydrolytic enzymes involved in various developmental and physiological processes such as seed germination, organ abscission, pod and anther dehiscence, and xylem cell formation. To date, no systematic analysis of polygalacturonase incorporating genome organization, gene structure, and expression profiling has been conducted in soybean (*Glycine max* var. Williams 82). In this study, we identified 112 *GmPG* genes from the soybean Wm82.a2v1 genome. These genes were classified into three groups, group I (105 genes), group II (5 genes), and group III (2 genes). Fifty-four pairs of duplicate paralogous genes were preferentially identified from duplicated regions of the soybean genome, which implied that long segmental duplications significantly contributed to the expansion of the *GmPG* gene family. Moreover, *GmPG* transcripts were analyzed in various tissues using RNA-seq data. The results showed the differential expression of 64 *GmPGs* in the tissue and partially redundant expression of some duplicate genes, while others showed functional diversity. These findings suggested that the *GmPGs* were retained by substantial subfunctionalization during the soybean evolutionary processes. Finally, evolutionary analysis based on single nucleotide polymorphisms (SNPs) in wild and cultivated soybeans revealed that 107 *GmPGs* had selected site(s), which indicated that these genes may have undergone strong selection during soybean domestication. Among them, one non-synonymous SNP of *GmPG031* affected floral development during selection, which was consistent with the results of RNA-seq and evolutionary analyses. Thus, our results contribute to the functional characterization of *GmPG* genes in soybean.

## Introduction

The plant cell wall is involved in many essential biological processes such as cell elongation, sloughing of cells at the root tip, fruit softening, fruit decay, pollen dehiscence and abscission of organs including leaves, floral parts, and fruits [1–3]. Therefore, the function and regulation of the cell wall have always piqued the interest of researchers [4]. Cell wall network assembly occurs through the action of cell wall hydrolytic enzymes, including polygalacturonase (PG), β-1,4-endoglucanases, pectate lyase, and pectin methyl esterase, which cleave the bonds between the polymers that make up the cell wall [5–6].

Among these hydrolytic enzymes, the PG belongs to one of the largest hydrolase families, which catalyze α-(1–4) linkages between D-galacturonic acid residues in homogalacturonan, causing cell separation [7]. Thus, PG activities are associated with a wide range of plant developmental programs such as seed germination, embryo development, organ abscission, pod and anther dehiscence, pollen grain maturation, xylem cell formation, and pollen tube growth [8–9]. Previous studies reported that PG was present in the endosperm cap of tomato seeds, and the activity of PG increased during seed germination [10–11]. The *Arabidopsis* exo-polygalacturonase gene *NIMNA* causes cell elongation defects in the early embryo and markedly reduces suspensor length [12]. Knocking out three *Arabidopsis PGs* led to the failure of pollen grain separation, and silique and anther dehiscence [13–14]. Over-expression of a PG in transgenic apple trees (*Malus domestica*) alters the leaf morphology and causes premature leaf shedding [15]. In tomato fruit, a correlation between endo-PG activity and softening was observed in some cultivars [16]. Moreover, the functions of *PGs* are not only restricted to plant developmental processes but also include wound responses and host-parasite interactions [17–18]. Thus, these findings illustrate that plant PG genes have extensive functional divergence.

Plant PGs are multifunctional proteins encoded by a large gene family. To date, genome-wide analyses of the PG gene family have focused mainly on annual herbaceous plants such as *Arabidopsis* and rice. The *Arabidopsis* and rice genomes contain 66 and 42 PG members, respectively, which are divided into three distinct groups [19]. Comparative analyses of this gene family will help understand the expansion and functional diversification of this large gene family. Soybean (*Glycine max*) is planted worldwide as an essential protein and oil crop; however, the functional characterization of PG genes in soybean has rarely been reported. In the present study, we conducted a detailed analysis of *GmPG* genes based on the genome Wm82.a2v1 including genome organization, gene structure, expression compendium, and selective effects of *GmPG* genes during soybean domestication. Our results may provide a subset of potential candidate PG genes for future engineering modification.

## Materials and Methods

### Sequence retrieval and phylogenetic analysis

To identify soybean PG gene family members, 66 Arabidopsis PG protein sequences were used to search the soybean genome database version 2 (http://www.phytozome.net/) using the TBLASTP program. The cut-off of E-value was 1e-10, and the score was 40%. Previous studies have shown that all PG proteins contained glycosyl hydrolase family 28 (GH28) domains (Kim et al., 2006) [19]. Thus, apart from sequence similarities, all collected soybean PG candidates were primarily analyzed using the protein families database (Pfam) to confirm the presence of GH28 domains in their protein structures. Multiple sequence alignments of the full-length protein sequences were performed by Clustal X (version 1.83) program [20]. The unrooted phylogenetic trees were constructed with MEGA 5.0 using the maximum-likelihood (ML) methods, and the bootstrap test was carried out with 1000 iterations [21]. The program MEME version 4.11.1 was used for the elucidation of motifs in 112 deduced soybean PG protein sequences (http://meme.sdsc.edu) [22].

### Genomic structure and gene duplication

Gene structure display server (GSDS) program was used to illustrate exon/intron organization of individual PG genes by comparison of the cDNA with its corresponding genomic DNA sequence from Phytozome (http://www.phytozome.net/) [23]. The identification of homologous chromosome segments resulting from whole-genome duplication events was accomplished as described in Schmutz et al. (2010) [24].

### Expression analysis of *GmPG* genes

Transcript data of the *GmPG* genes were downloaded from the Soybase database (http://soybase.org/). These were obtained from various tissues and developmental stages, including vegetative tissues (e.g., young leaf, root, and nodule), reproductive tissues (e.g., flower, one cm pod, pod shell of 10 and 14 days after flowering), and seeds from seven developmental stages (10, 14, 21, 25, 28, 35, and 42 days after flowering). All transcript data were analyzed with Cluster 3.0 [25], and the heat map was viewed using Java Treeview [26].

### Evolutionary analysis of *GmPG* genes

SNPs of the *GmPG* genes were downloaded from the NCBI dbSNP database based on the resequencing of 302 wild and cultivated soybean genomes [27]. Moreover, we analyzed the ratio of each SNP in wild and cultivated soybean populations. The SNP site with reverse distribution ratio in different types of soybean population was defined as a putative selective site throughout domestication.

### DNA polymorphism analysis

Candidate SNP of the soybean *PG031* gene was analyzed with a cleaved amplified polymorphic sequence (CAPS) marker, as follows. PCR using primers 5'- CTGTATCTCATTGGGTGATGGTAAC-3' and 5'- CCTGTTATTACGGGCTTGACG-3' amplified a 623-bp fragment from the genomic DNA. The amplified fragment from the PG031^289H^ allele had an Nsi I site containing the SNP; thus, it was digested into 217-bp and 406-bp fragments using this enzyme, whereas the PG031^289Y^ allele remained undigested. All genomic DNA of wild soybean and cultivars were provided by the Key Laboratory of Soybean Molecular Design Breeding of Northeast Institute of Geography and Agroecology.

### Gene expression model

A 1,786-bp fragment upstream of the *PG031* start codon was PCR amplified from the soybean genomic DNA using the following primers (5'-TAAAGTTCAAGGTGTTAGGAAGGTG-3' and 5'- ATTGTTTTTGTTTTTGTTTGTGGCA-3') for investigating *PG031* gene expression patterns. The PCR product was cloned into the Pst I/Nco I-digested pMDC1001G vector to generate the PG031_pro_:GUS expression construct. Plants were transformed with *Agrobacterium tumefaciens* strain LBA4404 using the floral dip method [28]. Positive transformants were selected on 1/2MS plates containing 50 mg·L^-1^ kanamycin. Flowers and siliques of T_2_ transgenic plants were subjected to GUS staining. GUS histochemical staining was performed by using 5-bromo-4-chloro-3-indolyl-b-D-glucuronide as substrates [29]. The tissues were decolored in 75% ethanol and images of GUS staining were recorded using a VHX digital microscope (Japan) or a Canon camera (Japan).

Semi-quantitative RT-PCR analysis was performed to further characterize the expression of soybean *PG031*. Total RNA was extracted from various tissues in cultivar DongNong50 (DN50, *PG031*^*289H*^-type) and Tokei 780 (TK780, *PG031*^*289Y*^-type) using the TRIzol method, and then subjected to reverse transcription using the SuperScriptTM III Reverse Transcriptase kit (Invitrogen, Carlsbad, CA, USA). RT-PCR specific primers were F: 5'- CTGTATCTCATTGGGTGATGGTAAC-3' and R: 5'-TTCAACGGCCTCTTCATTATC-3'; amplification of *β-tubulin* gene (Glyma.05G157300.1) was used as an internal control to normalize all data and cloned by primers (F: 5'-TCTTGGACAACGAAGCCATCT-3'; and R: 5'-TGGTGAGGGACGAAATGATCT-3').

### Generation of the *GmPG031* transgenic *Arabidopsis thaliana*

To obtain the transgenic *Arabidopsis* lines, the full length CDS of the *GmPG031*^*289H*^ and *GmPG031*^*289Y*^ genes were respectively amplified with the gene specific primers (5'-ATGAAGTTCACTATAATCACAATAT-3' and 5'-CTAGGCTGCACAAGTAGGAG-3'), and then linked with expression vector under the control of the strong constitutive CaMV35S promoter. The recombinant construct was transformed into Col-0 *Arabidopsis* as above, and transgenic lines were obtained by RT-PCR identification wherein the T_3_ lines were used for phenotypic analysis. Seedlings were transferred from 1/2 MS plates to the soil for growth in a greenhouse under controlled environment conditions (21–23°C, 200μmol photons m^-2^ s^-1^, 70% relative humidity, 16 h light/8 h dark cycles). Siliques were measured for at least three plants from each transgenic line.

## Results

### Sequence and phylogenetic relationships of soybean PG genes

We identified 112 genes encoding putative PG proteins in the soybean genome Wm82.a2v1 using the Phytozome database (S1 File). The detailed information of PG family genes in soybean including gene locus, location, and similarities to their *Arabidopsis* orthologs as well as amino acids are listed in Table 1. The 112 *GmPG* genes were distributed throughout the 20 soybean chromosomes and were numbered from GmPG001 to GmPG112 according to their localization. These identified PG genes in soybean encode proteins ranging from 67 to 882 amino acids (aa) with an average of 398 aa. Remarkably, in most cases, two or more soybean PG genes were found for every ortholog in *Arabidopsis*. We speculate that the presence of more *GmPG* genes may reflect a great need for complicated transcriptional regulation in this leguminous plant.

**Table 1:** Summary of PG family members in soybean.

Gene Symbol	Gene Locus	Chromosome	Start locus	End locus	strand	Arabidopsis ortholog locus	Identities	Extrons	Amino Acids	Alternative splicing events
GmPG001	Glyma.01G027900.1	Chr01	2912340	2915599	-	AT3G26610.1	62%	8	462	
GmPG002	Glyma.01G075000.1	Chr01	16645859	16649165	+	AT4G35670.1	43%	5	250	
GmPG003	Glyma.01G079800.1	Chr01	21340207	21343176	+	AT2G43890.1	62%	4	396	
GmPG004	Glyma.02G007600.1	Chr02	785557	790572	-	AT3G62110.1	68%	6	465	
GmPG005	Glyma.02G009300.1	Chr02	913657	916309	+	AT1G02460.1	70%	6	474	
GmPG006	Glyma.02G015600.1	Chr02	1395956	1400025	-	AT3G16850.1	60%	6	456	2
	Glyma.02G015600.2	Chr02	1395956	1400025	-	AT3G16850.1	54%	4	340	
GmPG007	Glyma.02G016300.1	Chr02	1460381	1463219	-	AT1G02790.1	45%	4	417	
GmPG008	Glyma.02G037300.1	Chr02	3462332	3465695	+	AT3G26610.1	63%	8	463	
GmPG009	Glyma.02G183000.1	Chr02	31506617	31510674	+	AT2G41850.1	57%	9	429	
GmPG010	Glyma.02G224700.1	Chr02	41216501	41217435	+	AT5G48140.1	46%	3	220	
GmPG011	Glyma.02G281600.1	Chr02	46391400	46394241	-	AT4G23820.1	73%	5	277	
GmPG012	Glyma.02G307000.1	Chr02	48146186	48147731	-	AT3G07840.1	46%	4	390	
GmPG013	Glyma.03G095900.1	Chr03	28312534	28315859	+	AT5G17200.1	45%	7	356	
GmPG014	Glyma.03G097500.1	Chr03	28506621	28512058	-	AT3G15720.1	44%	10	386	
GmPG015	Glyma.03G098700.1	Chr03	28682253	28684912	-	AT2G43870.1	63%	4	392	
GmPG016	Glyma.03G137700.1	Chr03	35379007	35382127	-	AT2G41850.1	54%	9	465	
GmPG017	Glyma.03G137800.1	Chr03	35385438	35390220	-	AT2G41850.1	47%	7	293	
GmPG018	Glyma.03G141000.1	Chr03	35715587	35715940	-	AT3G57790.1	53%	1	118	
GmPG019	Glyma.03G216800.1	Chr03	42081556	42084955	+	AT3G16850.1	61%	6	498	2
	Glyma.03G216800.2	Chr03	42081556	42084955	+	AT3G16850.1	61%	5	414	
GmPG020	Glyma.03G222500.1	Chr03	42538695	42542075	-	AT1G02460.1	71%	6	463	
GmPG021	Glyma.03G224600.1	Chr03	42691388	42695108	+	AT3G62110.1	68%	7	469	3
	Glyma.03G224600.2	Chr03	42691388	42695108	+	AT3G62110.1	68%	7	468	
	Glyma.03G224600.3	Chr03	42691388	42695108	+	AT3G62110.1	68%	6	466	
GmPG022	Glyma.04G143200.1	Chr04	25762308	25764069	+	AT3G07840.1	51%	4	390	
GmPG023	Glyma.04G157500.1	Chr04	38047214	38049008	-	AT3G07840.1	51%	4	390	
GmPG024	Glyma.04G157600.1	Chr04	38182883	38184627	-	AT3G07840.1	51%	4	390	
GmPG025	Glyma.04G157700.1	Chr04	38268620	38270375	-	AT3G07840.1	51%	4	390	
GmPG026	Glyma.05G005800.1	Chr05	508288	512615	+	AT5G14650.1	62%	6	440	
GmPG027	Glyma.05G133400.1	Chr05	32642084	32645811	+	AT1G48100.1	77%	6	491	3
	Glyma.05G133400.2	Chr05	32639275	32645721	+	AT1G48100.1	77%	6	491	
	Glyma.05G133400.3	Chr05	32641562	32645721	+	AT1G48100.1	77%	6	491	
GmPG028	Glyma.05G210900.1	Chr05	39264534	39267537	+	AT4G23500.1	75%	6	493	
GmPG029	Glyma.06G153900.1	Chr06	12545457	12549694	-	AT4G33440.1	67%	5	478	2
	Glyma.06G153900.2	Chr06	12545457	12549694	-	AT4G33440.1	72%	5	388	
GmPG030	Glyma.06G203000.1	Chr06	18962428	18964110	+	AT3G07820.1	54%	3	324	
GmPG031	Glyma.06G207300.1	Chr06	20011530	20013321	-	AT3G07840.1	51%	4	390	
GmPG032	Glyma.06G287800.1	Chr06	47663957	47664676	-	AT1G19170.1	81%	2	67	
GmPG033	Glyma.07G066900.1	Chr07	6023894	6029346	-	AT3G61490.3	73%	6	483	
GmPG034	Glyma.07G067000.1	Chr07	6032415	6036498	-	AT3G61490.2	70%	5	475	
GmPG035	Glyma.07G124100.1	Chr07	14707941	14715023	-	AT3G15720.1	46%	8	389	2
	Glyma.07G124100.2	Chr07	14707941	14715023	-	AT5G17200.1	41%	8	363	
GmPG036	Glyma.07G223500.1	Chr07	40029369	40032764	+	AT2G43870.1	63%	4	334	
GmPG037	Glyma.07G244200.1	Chr07	42376091	42379157	-	AT3G06770.2	66%	5	450	2
	Glyma.07G244200.2	Chr07	42376091	42379157	-	AT3G06770.2	66%	5	395	
GmPG038	Glyma.07G245100.1	Chr07	42439978	42441907	-	AT4G18180.1	44%	5	423	
GmPG039	Glyma.08G017300.1	Chr08	1401842	1404703	+	AT4G23500.1	74%	6	495	2
	Glyma.08G017300.2	Chr08	1402432	1404703	+	AT4G23500.1	74%	5	472	
GmPG040	Glyma.08G087900.1	Chr08	6661361	6667037	+	AT1G48100.1	77%	6	485	2
	Glyma.08G087900.2	Chr08	6663652	6667037	+	AT1G48100.1	77%	6	485	
GmPG041	Glyma.08G149300.1	Chr08	11441237	11445002	+	AT2G33160.1	48%	4	397	
GmPG042	Glyma.08G237800.1	Chr08	20272407	20273172	-	AT5G17200.1	41%	4	152	
GmPG043	Glyma.08G285400.1	Chr08	39493243	39498724	+	AT1G60590.1	63%	7	539	2
	Glyma.08G285400.2	Chr08	39493243	39498724	+	AT1G60590.1	73%	6	402	
GmPG044	Glyma.08G303200.1	Chr08	42128716	42133837	-	AT4G23820.1	77%	5	444	
GmPG045	Glyma.09G021100.1	Chr09	1663321	1674242	+	AT2G43870.1	58%	4	380	
GmPG046	Glyma.09G031600.1	Chr09	2592440	2595781	-	AT1G48100.1	72%	6	475	
GmPG047	Glyma.09G041000.1	Chr09	3405065	3408889	-	AT3G16850.1	64%	5	453	5
	Glyma.09G041000.2	Chr09	3405065	3408889	-	AT3G06770.2	63%	4	315	
	Glyma.09G041000.3	Chr09	3405065	3408889	-	AT3G06770.2	63%	4	315	
	Glyma.09G041000.4	Chr09	3405065	3408889	-	AT3G06770.2	65%	5	381	
	Glyma.09G041000.5	Chr09	3405065	3408889	-	AT3G06770.2	65%	5	381	
GmPG048	Glyma.09G041600.1	Chr09	3509893	3514058	-	AT1G02790.1	45%	5	423	
GmPG049	Glyma.09G071500.1	Chr09	7309029	7314952	+	AT1G19170.1	72%	5	495	
GmPG050	Glyma.09G085500.1	Chr09	10519361	10522202	-	AT2G43890.1	54%	4	395	
GmPG051	Glyma.09G132100.1	Chr09	32814091	32817773	-	AT3G61490.3	65%	6	477	
GmPG052	Glyma.09G224800.1	Chr09	44940935	44944621	-	AT3G07970.1	55%	9	442	
GmPG053	Glyma.09G233100.1	Chr09	45596647	45598043	-	AT3G15720.2	42%	6	246	
GmPG054	Glyma.09G256100.1	Chr09	47530401	47534434	-	AT3G61490.1	73%	6	485	
GmPG055	Glyma.10G010000.1	Chr10	935107	937794	+	AT1G02460.1	71%	6	476	
GmPG056	Glyma.10G016100.1	Chr10	1448652	1453370	-	AT3G16850.1	59%	6	457	2
	Glyma.10G016100.2	Chr10	1448652	1453370	-	AT3G16850.1	53%	4	341	
GmPG057	Glyma.10G016900.1	Chr10	1490555	1493810	-	AT1G02790.1	40%	8	378	
GmPG058	Glyma.10G088900.1	Chr10	11856714	11861649	-	AT3G07970.1	52%	9	439	
GmPG059	Glyma.10G103200.1	Chr10	21744662	21749301	+	AT2G41850.1	57%	10	478	
GmPG060	Glyma.10G135700.1	Chr10	36614685	36619793	+	AT3G48950.1	76%	2	181	
GmPG061	Glyma.10G138100.1	Chr10	37181749	37186039	+	AT3G62110.1	67%	6	465	4
	Glyma.10G138100.2	Chr10	37181793	37186039	+	AT3G62110.1	67%	4	354	
	Glyma.10G138100.3	Chr10	37181796	37185975	+	AT3G62110.1	67%	6	465	
	Glyma.10G138100.4	Chr10	37182820	37185975	+	AT3G62110.1	67%	4	354	
GmPG062	Glyma.10G144900.1	Chr10	37960875	37961222	+	AT1G02460.1	58%	1	116	
GmPG063	Glyma.10G230900.1	Chr10	46072235	46075548	-	AT3G61490.2	69%	6	467	
GmPG064	Glyma.10G231000.1	Chr10	46077416	46080227	-	AT3G48950.1	67%	6	481	
GmPG065	Glyma.10G231100.1	Chr10	46085150	46088062	-	AT3G48950.1	70%	6	472	
GmPG066	Glyma.11G157800.1	Chr11	14096320	14097228	+	AT3G14040.1	47%	3	240	
GmPG067	Glyma.12G003700.1	Chr12	294751	296671	+	AT3G15720.1	49%	8	361	
GmPG068	Glyma.12G012200.1	Chr12	895961	899249	+	AT3G07970.1	56%	9	441	
GmPG069	Glyma.12G173000.1	Chr12	32965198	32965973	+	AT5G27530.1	49%	2	88	
GmPG070	Glyma.13G080200.1	Chr13	18671747	18673041	+	AT3G07850.1	40%	4	190	
GmPG071	Glyma.13G111900.1	Chr13	22524471	22532116	+	AT1G19170.1	74%	5	492	4
	Glyma.13G111900.2	Chr13	22524562	22532116	+	AT1G19170.1	78%	3	278	
	Glyma.13G111900.3	Chr13	22526103	22532116	+	AT1G19170.1	80%	5	357	
	Glyma.13G111900.4	Chr13	22528863	22532116	+	AT1G19170.1	78%	3	278	
GmPG072	Glyma.13G364700.1	Chr13	45100703	45104460	-	AT5G17200.1	45%	9	402	
GmPG073	Glyma.14G006100.1	Chr14	487749	490051	+	AT3G07840.1	50%	4	393	6
	Glyma.14G006100.2	Chr14	484173	490050	+	AT3G07820.1	41%	3	387	
	Glyma.14G006100.3	Chr14	484736	490050	+	AT3G07840.1	51%	3	388	
	Glyma.14G006100.4	Chr14	484736	490050	+	AT3G07840.1	51%	3	384	
	Glyma.14G006100.5	Chr14	484736	490050	+	AT3G07840.1	51%	3	384	
	Glyma.14G006100.6	Chr14	484966	490050	+	AT3G07840.1	51%	3	384	
GmPG074	Glyma.14G032900.1	Chr14	2393952	2399559	+	AT4G23820.1	73%	5	447	
GmPG075	Glyma.14G044200.1	Chr14	3352725	3354812	+	AT3G15720.1	48%	8	348	
GmPG076	Glyma.14G149200.1	Chr14	32211624	32212810	-	AT4G18180.1	53%	5	227	
GmPG077	Glyma.14G152200.1	Chr14	32993506	32995974	-	AT1G02460.1	65%	6	294	
GmPG078	Glyma.14G191400.1	Chr14	45610980	45612600	+	AT3G07820.1	52%	4	387	
GmPG079	Glyma.15G008900.1	Chr15	704424	708837	+	AT5G17200.1	47%	10	426	
GmPG080	Glyma.15G009800.1	Chr15	753771	758859	-	AT1G80170.1	57%	10	459	6
	Glyma.15G009800.2	Chr15	753942	758859	-	AT1G80170.1	57%	10	458	
	Glyma.15G009800.3	Chr15	754231	758859	-	AT1G80170.1	60%	9	358	
	Glyma.15G009800.4	Chr15	755014	758859	-	AT1G80170.1	57%	10	459	
	Glyma.15G009800.5	Chr15	755199	758859	-	AT1G80170.1	57%	9	444	
	Glyma.15G009800.6	Chr15	755205	758859	-	AT1G80170.1	60%	8	343	
GmPG081	Glyma.15G127500.1	Chr15	10122886	10128876	+	AT2G43870.1	57%	4	409	
GmPG082	Glyma.15G136700.1	Chr15	11053772	11057169	-	AT1G48100.1	72%	6	480	2
	Glyma.15G136700.2	Chr15	11053772	11059037	-	AT1G48100.1	72%	6	480	
GmPG083	Glyma.15G146800.1	Chr15	12076031	12079018	-	AT3G16850.1	64%	5	472	2
	Glyma.15G146800.2	Chr15	12076031	12079129	-	AT3G16850.1	64%	5	453	
GmPG084	Glyma.15G151800.1	Chr15	12575444	12580908	+	AT1G02790.1	44%	5	423	
GmPG085	Glyma.15G151900.1	Chr15	12582175	12585280	+	AT1G02790.1	40%	7	407	
GmPG086	Glyma.15G179500.1	Chr15	17219937	17225941	+	AT1G19170.1	72%	5	490	
GmPG087	Glyma.15G196100.1	Chr15	22509381	22513175	+	AT2G43880.1	59%	4	402	
GmPG088	Glyma.15G196200.1	Chr15	22596267	22597074	+	AT2G43880.1	57%	2	67	
GmPG089	Glyma.15G269400.1	Chr15	50637589	50641041	+	AT2G33160.1	40%	5	365	
GmPG090	Glyma.15G275400.1	Chr15	51403963	51408843	-	AT3G07970.1	52%	9	440	2
	Glyma.15G275400.2	Chr15	51403963	51408843	-	AT3G07970.1	53%	8	364	
GmPG091	Glyma.16G033000.1	Chr16	3116063	3122058	-	AT3G61490.3	72%	6	492	2
	Glyma.16G033000.2	Chr16	3116063	3121227	-	AT3G61490.3	72%	6	490	
GmPG092	Glyma.16G118000.1	Chr16	26320844	26321558	-	AT5G14650.1	75%	2	87	
GmPG093	Glyma.16G179000.1	Chr16	33962757	33966062	-	AT3G61490.3	64%	6	478	
GmPG094	Glyma.17G029700.1	Chr17	2189547	2192494	+	AT3G06770.2	66%	5	450	
GmPG095	Glyma.17G047800.1	Chr17	3621562	3630290	-	AT1G19170.1	74%	5	493	
GmPG096	Glyma.17G209800.1	Chr17	34596230	34600725	-	AT4G35670.1	43%	7	270	
GmPG097	Glyma.18G065000.1	Chr18	5922025	5923204	+	AT1G78400.1	40%	3	151	
GmPG098	Glyma.18G116400.1	Chr18	14247625	14252903	+	AT4G23820.1	78%	5	443	4
	Glyma.18G116400.2	Chr18	14249367	14252903	+	AT4G23820.1	75%	4	308	
	Glyma.18G116400.3	Chr18	14249861	14252903	+	AT4G23820.1	75%	4	308	
	Glyma.18G116400.4	Chr18	14250185	14252903	+	AT4G23820.1	77%	4	334	
GmPG099	Glyma.18G137200.1	Chr18	20157804	20159757	-	AT3G07820.1	40%	7	277	
GmPG100	Glyma.18G139800.1	Chr18	21100714	21107360	+	AT1G23460.1	68%	12	882	
GmPG101	Glyma.18G139900.1	Chr18	21123201	21129260	+	AT1G60590.1	63%	7	539	4
	Glyma.18G139900.2	Chr18	21123201	21129260	+	AT1G10640.1	58%	6	495	
	Glyma.18G139900.3	Chr18	21123243	21129260	+	AT1G60590.1	72%	7	406	
	Glyma.18G139900.4	Chr18	21123973	21129260	+	AT1G60590.1	72%	7	406	
GmPG102	Glyma.18G236700.1	Chr18	52558809	52562720	+	AT3G61490.2	73%	6	485	
GmPG103	Glyma.19G006200.1	Chr19	590763	595425	+	AT5G14650.1	62%	6	444	
GmPG104	Glyma.19G140600.1	Chr19	40185556	40189614	-	AT2G41850.1	57%	10	524	
GmPG105	Glyma.19G143700.1	Chr19	40491825	40494810	-	AT3G57790.1	68%	3	467	
GmPG106	Glyma.19G213400.1	Chr19	46681858	46685501	+	AT3G16850.1	59%	6	483	2
	Glyma.19G213400.2	Chr19	46681858	46685501	+	AT3G62110.1	47%	6	432	
GmPG107	Glyma.19G219700.1	Chr19	47198635	47201809	-	AT1G02460.1	71%	6	463	2
	Glyma.19G219700.2	Chr19	47198635	47201809	-	AT1G02460.1	69%	5	448	
GmPG108	Glyma.19G221600.1	Chr19	47338816	47342912	+	AT3G62110.1	67%	7	469	3
	Glyma.19G221600.2	Chr19	47338861	47342854	+	AT3G62110.1	67%	6	466	
	Glyma.19G221600.3	Chr19	47338861	47342854	+	AT3G62110.1	67%	7	468	
GmPG109	Glyma.19G226500.1	Chr19	47823250	47825956	+	AT1G02460.1	65%	6	460	
GmPG110	Glyma.20G023200.1	Chr20	2459936	2463277	+	AT2G43870.1	60%	5	386	
GmPG111	Glyma.20G162700.1	Chr20	40031764	40032962	+	AT3G61490.1	60%	4	245	
GmPG112	Glyma.U016300.1	scaffold_21	2577809	2587232	-	AT4G35670.1	50%	7	403	4
	Glyma.U016300.2	scaffold_21	2577809	2587232	-	AT4G35670.1	51%	4	273	
	Glyma.U016300.3	scaffold_21	2577809	2587232	-	AT4G35670.1	49%	5	312	
	Glyma.U016300.4	scaffold_21	2577809	2587232	-	AT4G35670.1	49%	5	312	

**Fig 1:**
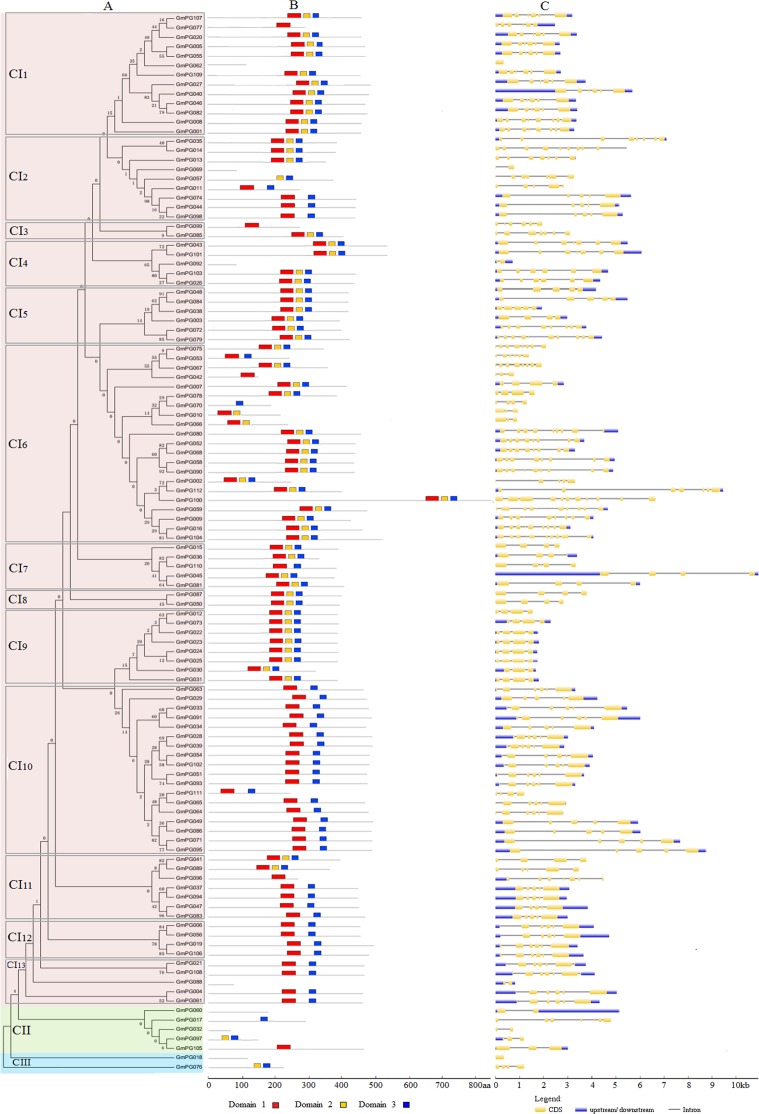
Phylogenetic relationships and gene structures of the *GmPG* genes. (A) Multiple alignments of 112 full-length amino acids of PG genes from soybean were executed by Clustal X ver. 1.83, and the phylogenetic tree was constructed using MEGA 5.0 by the maximum-likelihood (ML) method with 1,000 bootstrap replicates. Three distinct clusters (I to III) formed by the PGs are marked by red, green, and blue frames, respectively. (B) The main domains are highlighted by colored boxes. Introns are shown as lines. The sequence of the domains are shown in S1 Fig. (C) Exons and introns are represented by yellow boxes and gray lines, respectively. The sizes of exons and introns can be estimated using the scale at the bottom.

Subsequently, we constructed an unrooted tree to examine the phylogenetic relationships among *GmPG* genes using alignments of the full-length amino-acid sequences in their coding PG proteins (Fig 1A). The phylogenetic tree showed that the PGs formed three distinct clades (red, green, and blue boxes) with 100% bootstrap support. In the tree, the PG genes in the red, blue, and green clades were termed cluster I, II, and III PGs, respectively. Soybean classes I, II, and III PG genes contained 105, 5, and 2 members, respectively. Class I was divided into subfamilies CI_1_ to CI_13_ according to the most recent common ancestor (MRCA) of soybean. Phylogenetic tree topology revealed that 36 *GmPG* gene pairs located at the terminal nodes shared a sequence similarity of 52%~99%. This implied that these genes were homologous genes that diverged by gene duplication.

Additionally, through multiple alignment analysis, we also discovered the features of the homologous domain sequence and the frequency of the amino-acids at each position on the GmPG domains. As shown in S1 Fig, three distinct motifs, which are the main domains of the PG family, were identified. Among the 112 *GmPG* genes, motifs 1 and 3 were present in most of the GmPG family members (Fig 1B). Noticeably, some specific motifs were present in PGs; for instance, domains 1 and 3 of subfamily CI_10_-CI_13_, domains 1 and 2 of Cluster II, and domains 2 and 3 of Cluster III. However, some of these were PG fragments and had no domains such as *GmPG062*, *GmPG069*, *GmPG092*, *and GmPG018*, which were considered to be pseudogenes in the study. These results suggested that these motifs might confer unique functional roles to soybean PG proteins.

### Gene structure and gene duplication of soybean PG genes

To determine the numbers and positions of exons and introns within each soybean PG gene, we used the full-length cDNA sequences with the corresponding genomic DNA sequences. We observed that introns disrupted most of the coding sequences of the PGs. By contrast, two genes (*GmPG062 and 018)* had no introns in their coding region (Fig 1C). The remaining genes had up to 11 introns based on their relative positions. These results supported the argument that gene structural diversity was a possible explanation to the evolution of multigene families [30]. However, gene structures not only showed extreme similarity in most of the closely related *GmPG* members at the same node, but the position and length of intron were almost completely conserved. This high level of similarity suggested that these genes arose from a recent duplication event.

**Fig 2:**
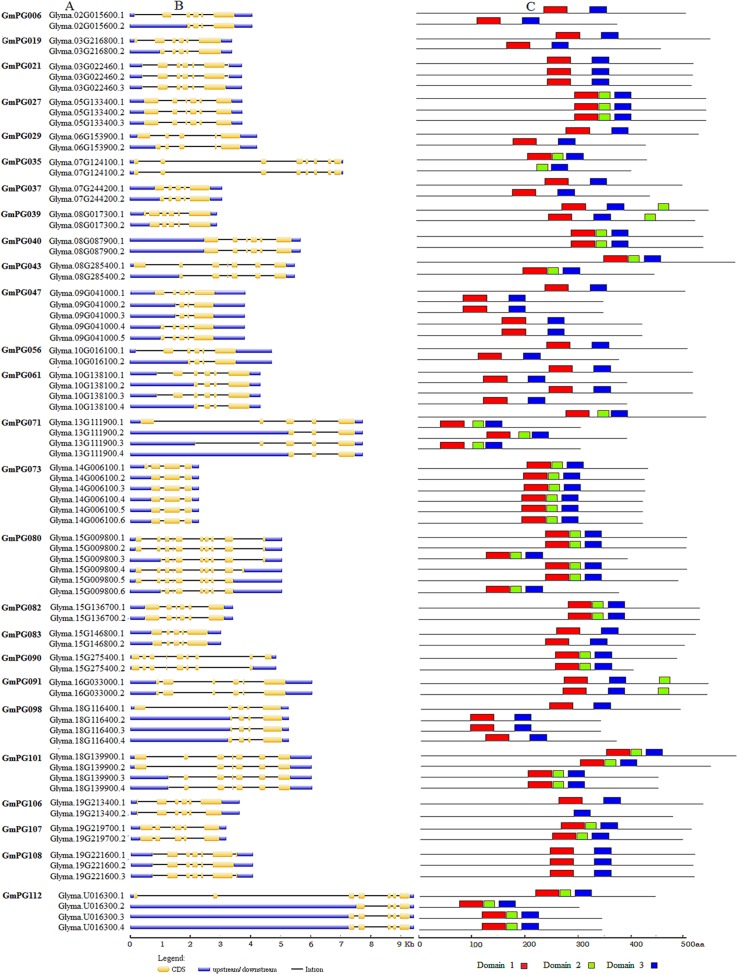
Alternative splicing of *GmPG* genes in the soybean genome. (A) The 26 identified *GmPGs* contained alternative structures. (B) Among these genes, 21 genes underwent extension, shortening or deletion of exon sequences, three underwent 5'-UTR events, and three had competing 5'/3'-UTR events. (C) The main domains are indicated by colored boxes.

Furthermore, 26 *GmPG* genes contained two to six alternative structures that had undergone alternative splicing (AS) and thus produced a variety of transcripts from a single gene (Fig 2). Among these genes, 21 genes underwent extension, shortening or deletion of exon sequences, three underwent 5'-UTR events, and three had competing 5'/3'-UTR events. Interestingly, *GmPG073* and *GmPG080* exhibited six alternative types of splicing by 5/3' alternative splice and extending or shortening the exon. The remaining three alternative splice events (*GmPG040*, *GmPG082*, and *GmPG091*) occurred in the 5'-UTR region without affecting the coding frame, which indicated that they created a variety of UTRs that may play a key role in gene regulation. Besides, some of the AS events resulted in a variety of domain insertions and/or deletions in the corresponding coding region. For instance, the exon deletion in *GmPG035* resulted in the deletion of domain 1. The AS events enriched gene structures and might be a consequence of function diversity.

**Fig 3:**
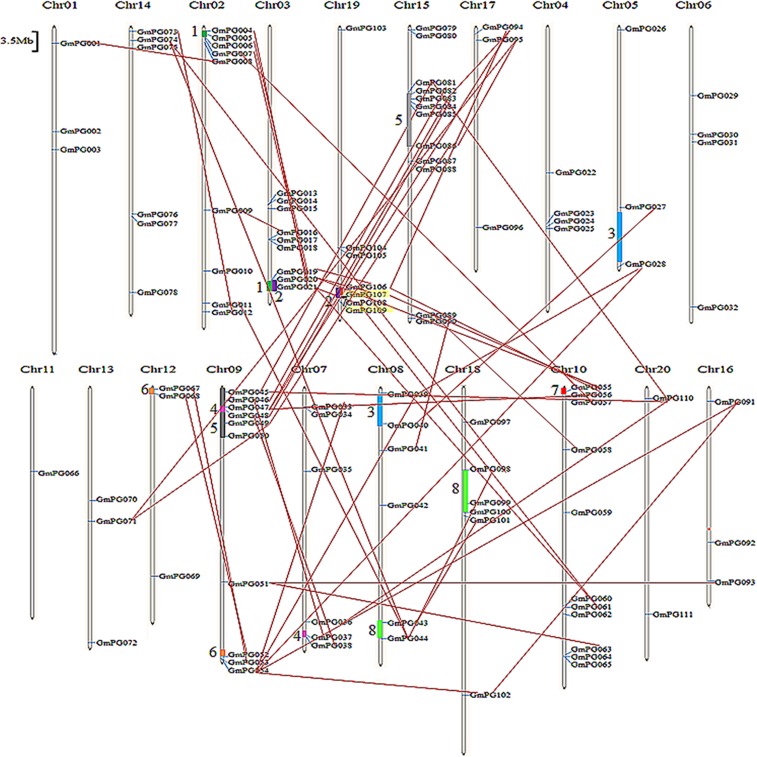
Chromosomal locations and region duplication of *GmPG* genes. A total of 112 *GmPG* genes are mapped to the 20 chromosomes (Chr) on the basis of JGI soybean Genome version 7.0. Each pair of duplicated PG genes is connected with a red line, generating a total of 54 gene pairs. Segmental duplicated homologous blocks are indicated with the same color bar, a total of 8 predicted duplication regions. Tandemly duplicated genes are indicated with yellow box. The chromosome number is indicated above each chromosome. The scale is in megabases (Mb). Scale represents a 3.5 Mb chromosomal distance.

Moreover, gene duplication occurs throughout plant evolution, thereby contributing to the establishment of gene-family expansion and new gene functions [31–32]. Paralogous segments created by this whole-genome duplication event were identified in previous analyses of the soybean genome [24]. Fifty-four duplicate pairs relative to the corresponding duplicate blocks are illustrated in Fig 3 and S1 Table, including 53 segmental duplications and one tandem duplication. Twenty-three PG pairs were clearly located in collinear regions and formed eight blocks. For example, *GmPG004*, *005*, *006* showed extensive collinearity corresponding to the duplicated regions *GmPG021*, *020* and *019*; *GmPG045*, *046*, *047*, *048* were duplicated by *GmPG081*, *082*, *083*, *084*, and *086*, which were collinearly arranged. These results indicated that these eight blocks were derived from large-scale duplications of their associated blocks. Moreover, *GmPG033* and *GmPG039* were flanked by *GmPG054*, whereas *GmPG056*, *083*, and *094* were flanked by *GmPG047*, indicating that these are products of a block duplication. All of these findings clearly suggest that several members of the PG family were derived from large-scale duplication events.

### Differential expression profile of soybean PG genes

**Fig 4:**
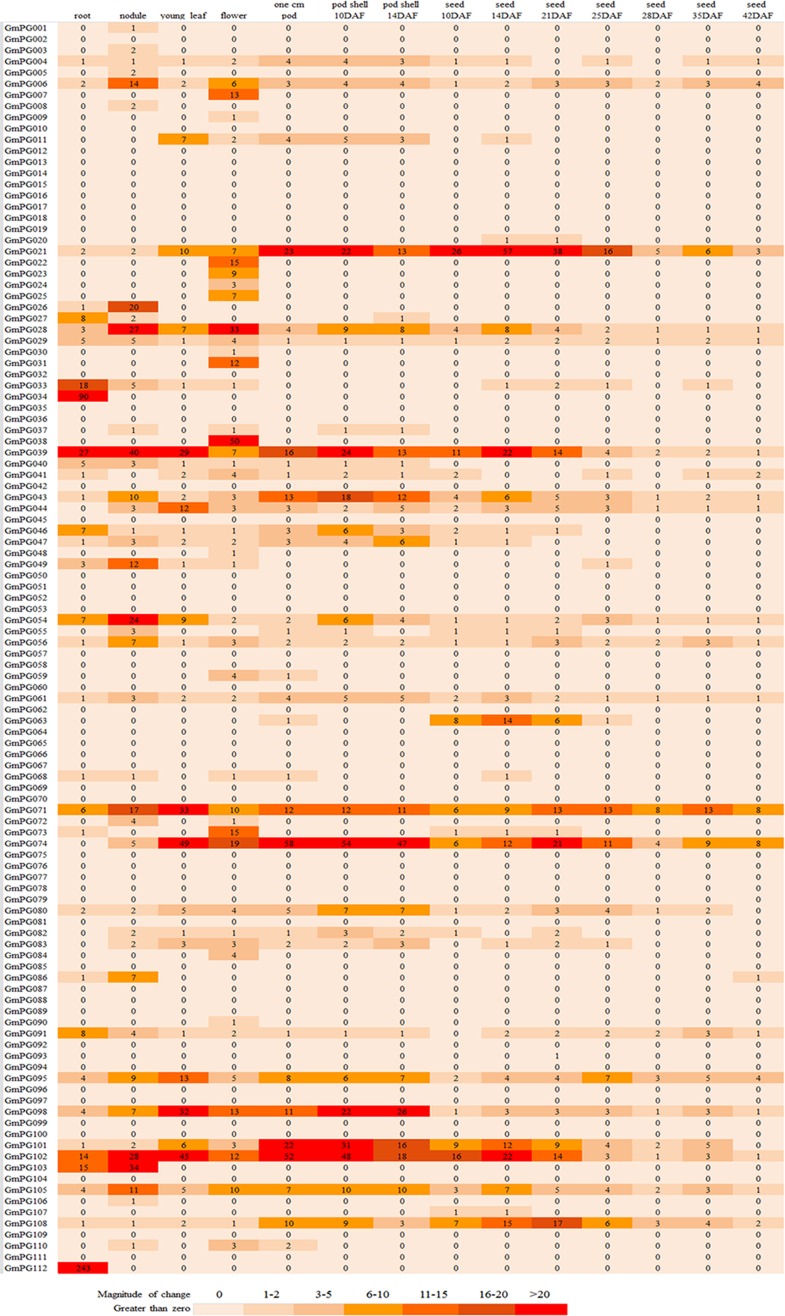
Expression profile of *GmPG* genes in different tissues. The numbers in the expression profile are normalized data, which were calculated as reads, normalization of the raw data. All data were selected from the SoyBase databases.

**Fig 5:**
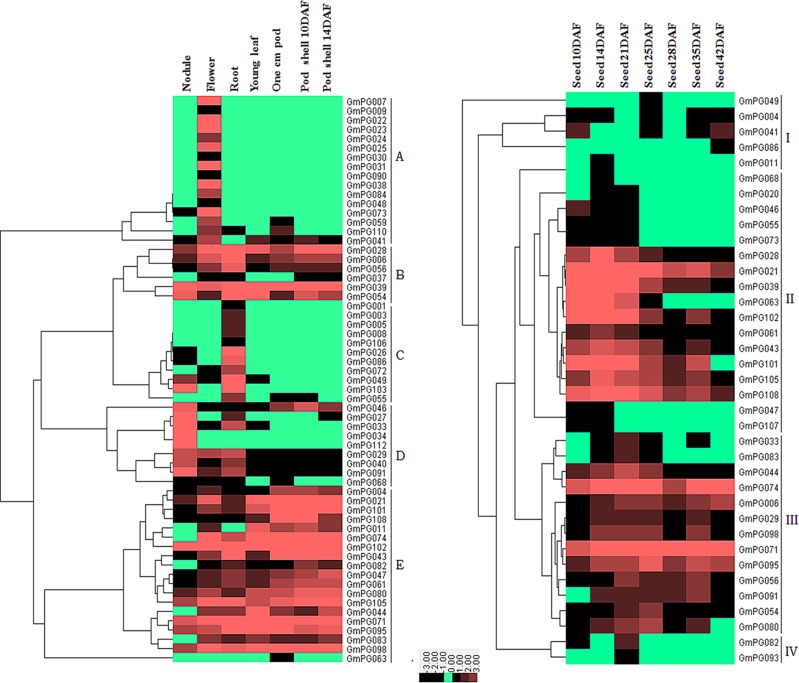
Heat map of 64 expressed *GmPG* genes in different tissues. (A) Heat map showing hierarchical clustering of 61 expressed *GmPG* genes among various tissues analyzed. (B) Heat map showing hierarchical clustering of 37 expressed *GmPG* genes during the development of soybean seeds.

Gene expression pattern may provide important clues to gene function [33]. We therefore obtained the previously publicly-available RNA-seq data across six soybean tissues and seven seed developmental stages [34]. RNA-seq data analysis showed that 64 *GmPG* genes had sequence reads in at least one tissue (Fig 4). These genes were clustered into five groups (A-E) and four groups (I-IV) based on their expression profiles in the soybean tissues (except seeds) and the expression patterns during seven soybean seed development stages (Fig 5). These genes in clusters A-E were mainly expressed in flower, root/flower/pod, root, nodule, and pod/leaf/root/flower, respectively. Further, most genes of cluster II were highly expressed at the earlier stage of seed development. Most genes of cluster III were expressed during the whole soybean seed development process. Thus, the wide expression of these genes illustrated that soybean PG genes had extensive functional divergence. Moreover, many genes showed a distinct tissue-specific expression pattern, suggesting specific roles in particular stages of development. For instance, six genes (*GmPG038*, *022*, *007*, *031*, *023*, and *025*) displayed specifically expression in the flower of soybean. Three genes (*GmPG026*, *072*, and *086*) had a significantly transcript accumulation in the root. *GmPG034* and *GmPG112* were highly expressed in nodule. Four genes such as *GmPG021*, *GmPG039*, *GmPG063*, and *GmPG102* were primarily expressed at the seed development stage. Besides, for most of the members of the subfamily CI_10_-CI_13_, *GmPGs* accumulated transcripts in various tissues. Subfamily CI_9_ *GmPGs* were mainly expressed in the flower. For some *GmPG* genes, there was no domain 1 in their protein, which showed a relative low expression level.

Duplicate genes may have different evolutionary fates: nonfunctionalization, neofunctionalization, or subfunctionalization, which may be indicated by divergence in expression patterns [35]. In this study, we investigated the functional redundancy of the *GmPG* genes with high proportion of segmental/tandem duplications. Of the 54 homologous pairs of *GmPG* genes, 16 paralogous pairs shared similar expression patterns; for example, *GmPG001/008*, *GmPG004/021*, *GmPG043/101*, *GmPG028/054*, and *GmPG048/084*, etc. In contrast, the expression patterns of the 35 duplicate genes were partially redundant, from which distinct pattern shifts were discerned. For example, *GmPG112* gene was mainly expressed in the nodule, whereas its duplicate counterpart *GmPG002* gene was hardly expressed in the tissues. *GmPG071* showed a high expression level in seeds, but its duplicate counterpart *GmPG049* showed a relatively low expression level. *GmPG006* extended to broader expression patterns in tissues while its duplicate counterpart *GmPG019* had no expression. The other three gene pairs barely had any corresponding data in various tissues. These findings suggested that expression profiles had diverged substantially after gene duplication. Consequently, we speculate that *GmPGs* have been retained by substantial subfunctionalization during the soybean evolutionary processes.

### Artificial selection analysis for *GmPGs* during soybean domestication

Cultivated soybean was domesticated from wild soybean (*Glycine soja*) in China 5,000 years ago. Therefore, large numbers of protein-coding genes underwent selection during soybean domestication. Here, we present a survey of the selection effects using 112 soybean PG genes during soybean domestication based on the sequence diversity analysis in soybean populations including 62 *G*. *soja*, 130 landraces, and 110 improved cultivars [27]. We determined that 1726 selected SNPs existed in the 107 soybean PG genes, including 452, 1,110, 88, and 66 SNPs in exon, intron, 5'UTR, and 3'UTR, respectively (S2 Table). Moreover, most of them had more than one selected site. These results suggested that the sites had experienced selection during soybean domestication and improvement. The SNPs were distributed throughout the 20 soybean chromosomes, mainly Chr09, Chr14, Chr15, and Chr19 (S2 Fig). Among these, 549 sites were significantly decreased from wild soybeans to landraces and to cultivars. On the contrary, the genetic diversity of 542 sites increased sharply in cultivars compared with that of landraces or wild soybeans. These results suggested that many sites probably might have been artificially selected to meet human needs or adapt to their environment. In addition, the reverse distribution of SNP in different evolutionary type of soybeans was defined as strong selected site [30]. 594 strong selected sites were identified and located in 91 *GmPGs* (S3 Table). So these PG genes with one or more type of reverse distribution were assumed to undergo an artificial selection during soybean domestication.

Domestication involves the genetic modification of functional units. Therefore, we found that 86 *GmPGs* had nonsynonymous selected site(s) in their coding sequence (CDS), and 42 of them had sequence reads in at least one tissue (S4 Table). Interestingly, nonsynonymous selected site(s) of six *PG*s (*PG007*, *011*, *022*, *031*, *034*, and *107*) had a single haplotype in wild-soybeans and various in cultivars. These genes were mainly expressed in the flower, flower/leaf/pod, flower, flower, nodule, and seed, respectively. Moreover, nonsynonymous selected site(s) of the three *PG*s (*PG039*, *54*, and *74*) had a single haplotype in landraces and various in wild-soybeans. These genes had a high transcript accumulation in various tissues. These selected sites may have caused functional changes in the corresponding *GmPGs* during soybean domestication.

Recently, more evidence demonstrated the significance of PG genes in flower development [13–14]. In an attempt to address whether the nonsynonymous selected site(s) may influence flower development, *PG007*, *11*, *22*, *31*, *39*, *54*, and 74 above mentioned were selected as our study aim. Because more than two selected sites were identified in *PG007*, *11*, *39*, *54*, and 74, we barely obtained a single haplotype using one SNP. Moreover, the nonsynonymous SNP of *PG022* showed lower diversity than the nonsynonymous SNP of *PG031* between 17 wild soybean and 14 cultivars according to Lam’s resequencing data [36]. Finally, we selected a specific SNP from *GmPG031* to explore the preliminary role of soybean PG genes during soybean domestication.

### Allelic variation of soybean *PG031* gene in wild and cultivated soybean populations

**Fig 6:**
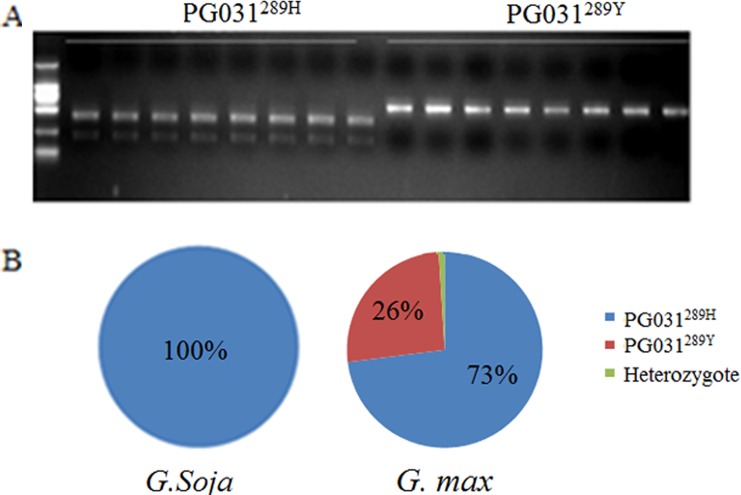
CAPS marker used to detect the SNP in *PG031*. (A) A fragment of 623 bp harboring the SNP can be digested by Nsi I in the *PG031*^*289H*^, but not in the *PG031*^*289Y*^. (B) Genotypic constitutions at the CAPS marker for 815 soybean population

Compared to the Williams 82 reference sequence, the SNP above was detected at the 867th nucleotide from the start codon of the *PG031* gene. Moreover, the SNP caused an AA substitution from (H) in wild soybean to (Y) in cultivars at 289th amino acid of the PG031 protein. The protein was identified by CAPS-specific primers, which amplified a 623-bp fragment containing the SNP, which was then digested using Nsi I into 217-bp and 406-bp fragments of the *PG031*^*289H*^-type, but not of the *PG031*^*289Y*^-type (Fig 6A).

To elucidate polymorphism of the SNP alleles, we conducted genotype analysis using the minicore collection of the Chinese soybean landraces. Our results showed that the SNP locus was *PG031*^*289H*^ in all of the *G*. *soja* population; in cultivars, the SNP was mainly *PG031*^*289H*^, whereas *PG031*^*289Y*^ shared 26% (Fig 6B and S5 Table). This was consistent with the results presented in S4 Table. Therefore, we speculated that the SNP substitution of *PG031* might have resulted in a loss of function or gain in function.

### *PG031* involved in flower development

**Fig 7:**
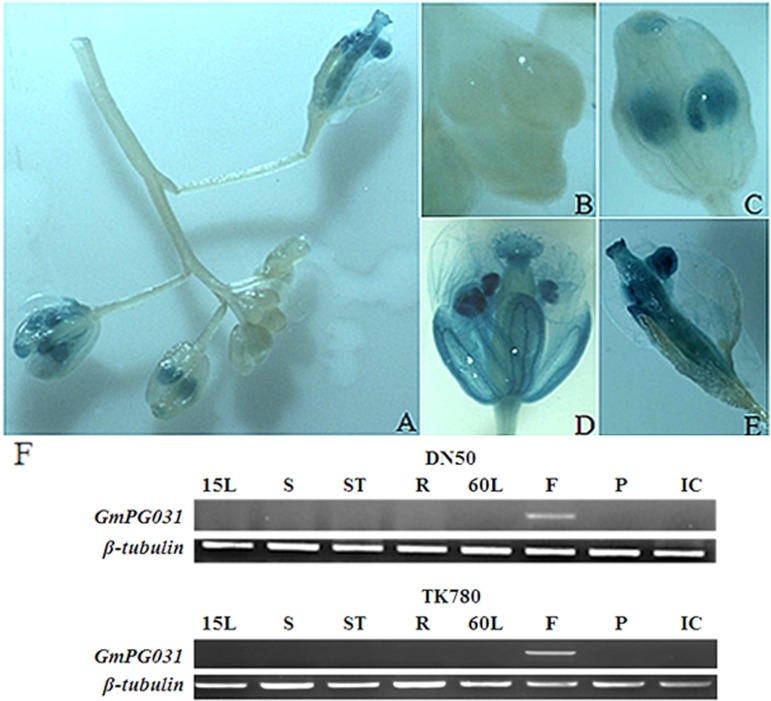
*GmPG031* expressed in flowers and siliques. (A) GUS staining of GmPG031pro::GUS transgenic plants flowers. (B-E) Close-up images of transgenic plants flowers in A. (F) RT-PCR identification of *GmPG031* in different tissues including 15-d-old leaf (15L),60-d-old leaf (60L), stems (S), stem tip (ST), roots (R), flowers(F), pod (P) and **i**mmature embryos (IC) in soybean DN50 and TK780. *β-tubulin* was used as an internal control.

We investigated the specificity of *PG031* expression in a tissue to elucidate the role of the soybean *PG031* gene in flower. For this, we fused a 1,786-bp fragment (upstream of the *GmPG031* translation initiation site) to the GUS reporter gene and introduced it into *Arabidopsis* via *Agrobacterium*-mediated transformation. The transgenic seedlings of T_3_ generation were subjected to GUS staining for activity analysis. We observed strong GUS activity in the flowers of *PG031*-GUS plants (Fig 7A). Specifically, strong activity appeared in both pollen and tube but not in the buds (Fig 7B–7E). Moreover, RT-PCR amplification from DN50 (*PG031*^*289H*^-type) and TK780 (*PG031*^*289Y*^-type) showed a significant increase in *PG031* transcripts in the flower, but there were no transcripts in the leaf/stem/seed (Fig 7F). The *PG031* gene showed flower-specific expression pattern, which was in agreement with the RNA-seq profile (Fig 4). Thus, we speculate that the *PG031* gene might play a role in flower development.

### *GmPG031* affected flowers and siliques development

**Fig 8:**
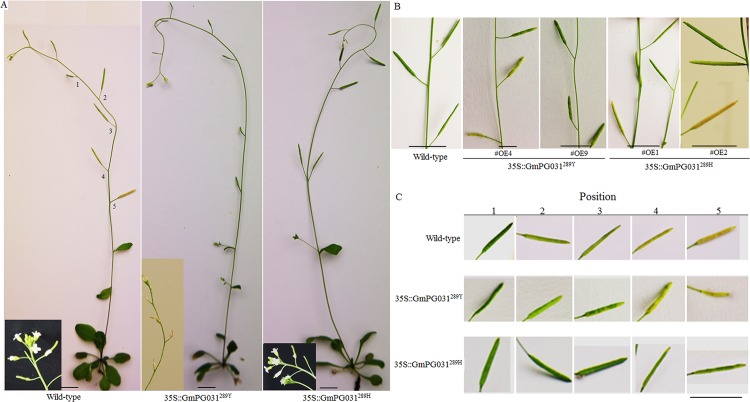
*GmPG031* gene influences flower and silique development. (A) Phenotypes of WT, 35S::GmPG031^289Y^ and 35S::GmPG031^289H^ plants. Some of 35S::GmPG031^289Y^ transgenic plants have dead inflorescence. (B) 35S::GmPG031^289Y^ siliques appeared less full, but WT and 35S::GmPG031^289H^ siliques relatively. (C) A series of siliques at different positions were compared between transgenic and wild-type plants (Stage 15). Different positions are shown in A.

**Fig 9:**
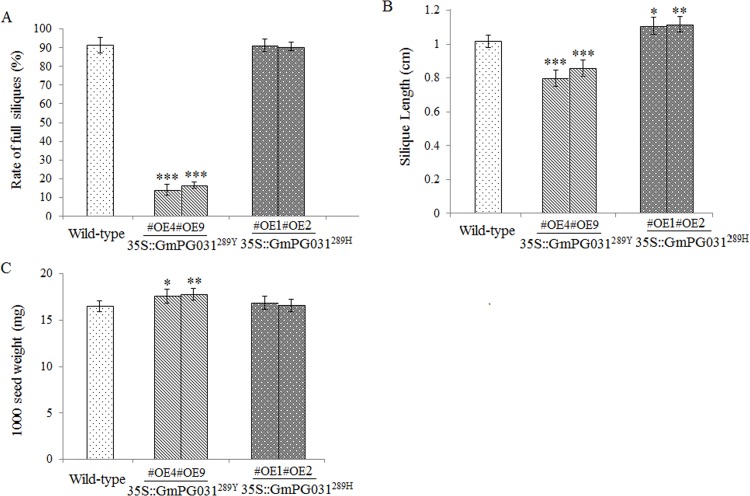
Identification of siliques in G*mPG031* transgenic and wild-type plants. (A) The rate of full silique in GmPG031^289H^ plants was higher than that in GmPG031^289Y^ transgenic *Arabidopsis*. (B) Silique length of WT, 35S::GmPG031^289Y^ and 35S::GmPG031^289H^ plants. (C) The 1,000-seed weight of 35S::GmPG031^289Y^ was significantly higher than that of 35S::GmPG031^289H^ plants. Stars indicate significantly different groups (P < 0.05, Tukey test).

To understand the biological role of soybean *PG031* in flower, the *GmPG031*^*289H*^ and *GmPG031*^*289Y*^ genes were introduced in *Arabidopsis* under the control of CaMV35 promoter. As shown in Fig 8A, growth status seemed similar between GmPG031^289H^ and GmPG031^289Y^ transgenic plants. When they entered into the flowering stage, some of the inflorescences died in 35S::GmPG031^289Y^ plants, but survived in WT and 35S::GmPG031^289H^. In addition, 35S::GmPG031^289Y^ siliques had survived but appeared less full, curved shorter, and attained early maturity, whereas the WT and 35S::GmPG031^289H^ siliques developed at a normal rate (Fig 8B and 8C). The rate of full silique in 35S::GmPG031^289H^ plants was significantly higher than that of 35S::GmPG031^289Y^ (Fig 9A). The silique length of 35S::GmPG031^289H^ plants was significantly longer than that of 35S::GmPG031^289Y^ and the wild-type (Fig 9B). In addition, the selected SNP of the *GmPG031* gene affected seed number and weight. Because the siliques showed less full in the GmPG031^289Y^ transgenic *Arabidopsis*, the number of seeds was lower than that of GmPG031^289H^ plants. However, the 1,000-seed weight of 35S::GmPG031^289Y^ was significantly higher than that of 35S::GmPG031^289H^ (Fig 9C, P-value<0.05).

Seed weight is an important trait of soybean domestication. Our phenotypic observations on seed weight in transgenic *Arabidopsis* has proven that the detected SNP occurred during the domestication of wild soybean and is responsible for the difference in seed weight between wild and cultivated soybeans.

## Discussion

PG is one of the major enzymes involved in pectin disassembly by biochemically catalyzing the hydrolytic cleavage of a (1–4) galacturonan [37–38]. Pectin is one of the major components of the primary cell wall, whose disassembling leads to cell wall separation [39–40]. Therefore, PG was suggested to play important roles in many stages of plant development, particularly in various cell separation processes [41–42].

PGs belong to a large gene family in plant genomes. A recent study identified 66, 75, and 44 *PG* genes from *Arabidopsis*, *Populus*, and rice genomes, respectively, which were divided into three classes [43]. In this study, we found that the 112 PGs from the soybean genomes could be classified into three distinct groups (Fig 1). Compared to *Arabidopsis*, *Populus*, and rice PGs, the soybean genome contained the highest number of PG members. Therefore, multiple soybean genes may be required to maintain their biological functions of adapting to more complex organ systems and structures. Furthermore, RNA-seq showed that 64 *GmPGs* had sequence reads in at least one tissue. Approximately 48 of these 112 (43%) *GmPGs* showed transcript accumulation in flower tissues, 42 (38%) showed the transcript accumulation in roots, 30 (27%) showed the transcript accumulation in leaves, 34 (30%) showed the transcript accumulation in pods, and 37 (33%) showed the highest transcript accumulation in seeds (Figs 4 and 5). Variations in the expression levels of *GmPGs* indicate that multiple *GmPGs* are necessary for the complicated transcriptional regulations during the development of all organs or tissues in soybean.

To our knowledge, the primary causes of gene-family expansion include segmental duplication, tandem duplication, and transposition events. Combined with the soybean genome, *GmPG* genes were generated mainly through segmental duplication events and non-randomly distributed across all 20 chromosomes. Moreover, one gene of some duplicate pairs showed a relatively low expression level, whereas the other showed functional diversity, which may lead to neofunctionalization or subfunctionalization. These results support the theory that segmental duplication events may widely distribute duplicated genes across the genome, and could lead to the loss of many functional redundant genes to avoid fitness cost [44–45]. Moreover, AS contributes to gene-family diversity, which generates various gene isoforms for differential expression.

With the rapid development of next-generation sequencing technology, powerful genomic approaches have been used to screen for selective sweeps or genes at a genome-wide level [36, 46]. Thus far, large numbers of protein-coding genes undergoing selection during plant domestication have been identified from soybean such as *GmCupin* genes, *GmPLC* genes [30, 47]. Similarly, PGs are involved in diverse biological processes. Therefore, we comprehensively investigated SNPs in soybean PG genes using 302 resequenced soybean accessions in this study. Our results revealed that most of the *GmPGs* underwent strong natural selection during soybean domestication, with some exhibiting a certain degree of variation. Whether these SNPs confer unique functional roles remains to be further investigated.

To determine whether the identified SNP plays a putative functional role in plant development, one SNP of *GmPG031* gene was selected as a preliminary candidate according to the RNA-seq data and evolutionary analysis results. Furthermore, we performed genotype, promoter pattern, and transgene expression analysis. Genotype analysis indicated that the SNP of the *GmPG031* gene underwent strong selection during soybean domestication. Promoter and over-expression analysese indicated that the selected SNP apparently affected floral development (Figs 6 and 7). Thus, our study suggested that the differential selection patterns may be associated with their functions. Although there is a lack of experimental evidence for the involvement of PG gene in soybean development or organogenesis, understanding the role of PGs in domestication may help answer fundamental biological questions and enhance our ability to engineer crops.

## Supporting Information

S1 Fig Conserved domains across PG proteins in soybean. (TIF)

S2 Fig Distribution of the selected SNPs in soybean chromosomes. (TIF)

S1 File Transcript sequences of soybean PG genes. (TXT)

S1 Table Gene duplication and gene blocks of the *GmPG* genes. (XLSX)

S2 Table Selected sites of soybean PG genes during soybean domestication. (XLSX)

S3 Table Strong selected sites of *GmPGs* during soybean domestication. (XLSX)

S4 Table Nonsynonymous selected sites of soybean PG genes during soybean domestication. (XLSX)

S5 Table Polymorphism analysis of candidate SNP in soybean *PG031* gene. (XLS)
