# Supplementary figures and images for: A Global Analysis of the Polygalacturonase Gene Family in Soybean (Glycine max)
Source: PLoS One. 2016 Sep 22;11(9):e0163012. doi: 10.1371/journal.pone.0163012 (PMC5033254; doi:10.1371/journal.pone.0163012)

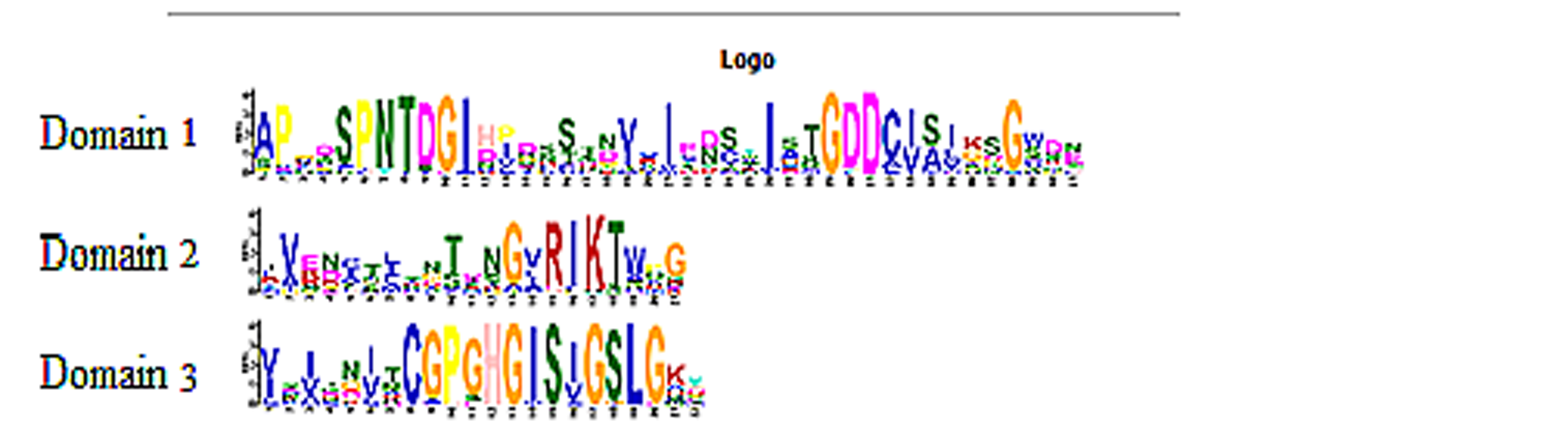

Supplement: S1 Fig — (TIF) [file pone.0163012.s001.tif]
